# Effectiveness of introgression of resistance loci for Gibberella ear rot from two European flint landraces into adapted elite maize (*Zea mays* L.)

**DOI:** 10.1371/journal.pone.0292095

**Published:** 2023-09-27

**Authors:** Félicien Akohoue, Silvia Koch, Bärbel Lieberherr, Bettina Kessel, Thomas Presterl, Thomas Miedaner

**Affiliations:** 1 State Plant Breeding Institute, University of Hohenheim, Stuttgart, Germany; 2 Kleinwanzlebener Saatzucht (KWS) KWS SAAT SE & Co. KGaA, Einbeck, Germany; University of Guelph, CANADA

## Abstract

European flint landraces are a major class of maize possessing favorable alleles for improving host resistance to Gibberella ear rot (GER) disease which reduces yield and contaminates the grains with mycotoxins. However, the incorporation of these landraces into breeding programs requires a clear understanding of the effectiveness of their introgression into elite materials. We evaluated 15 pre-selected doubled haploid (DH) lines from two European flint landraces, “Kemater Landmais Gelb” (KE) and “Petkuser Ferdinand Rot” (PE), together with two adapted elite flint lines and seven standard lines for GER severity as the main trait, and several adaptation traits (plant height, days to silking, seed-set, plant vigor) across four environments. From this evaluation, three KE DH lines and one PE DH line, with the lowest GER severity, were selected and used as donor parents that were crossed with the two adapted and GER susceptible flint lines (Flint1 and Flint2) to develop six bi-parental DH populations with 34−145 DH lines each. Each DH population was evaluated across two locations. Correlations between GER severity, which was the target trait, and adaptation traits were weak (−0.02 to 0.19). GER severity of lines from PE landrace was on average 2-fold higher than lines from KE landrace, indicating a clear superiority of the KE landrace lines. Mean GER severity of the DH populations was 39.4−61.0% lower than the adapted elite flint lines. All KE-derived DH populations were on average more resistant (27.0−36.7%) than the PE-derived population (51.0%). Highly resistant lines (1.3−5.2%) were found in all of the populations, suggesting that the DH populations can be successfully integrated into elite breeding programs. The findings demonstrate that selected KE landrace lines used as donors were effective in improving GER resistance of the adapted elite inbreds.

## Introduction

Maize (*Zea mays* L.) is the most important cereal crop before wheat (*Triticum aestivum* L.) and rice (*Oryza* sp.) for worldwide production. In Northwestern (NW) Europe, maize cultivation started on a large scale with the upcoming of hybrid cultivars in the 1960s. However, in the 500 years since Columbus’ voyages, a great array of landraces were cultivated in NW Europe which descended mainly from Northern flints introduced from Northeastern United States of America (USA) or Canada in the 16^th^ and 17^th^ century [1]. These landraces evolved upon a long time and adapted in the allogamous crop by selection for specific agro-climatic conditions. They are highly heterozygous and show a high within landrace genetic variance as indicated, for example, for ergot resistance in rye populations [2] and for agronomic traits in maize [3]. This was a motivation to produce doubled haploid (DH) line libraries of selected landraces [4] and to test them for adaptation and target traits.

Unfortunately, all these landraces disappeared with the advent of hybrid cultivars [5], however, they may contain favorable alleles not present in elite gene pools. The flint gene pool is still indispensable for maize cultivation in NW Europe because of its earliness and cold tolerance [6], but relies on only a few first-cycle founder lines extracted from a handful landraces [3]. Molecular studies show that only a tiny fraction of the landraces have been incorporated into modern flint elite lines [7]. Lines derived from landraces, however, have a yield gap to the elite material and are often lacking important adaptation traits like earliness, lodging tolerance, resistance to corn smut (*Ustilago maydis*) and viruses like sugar cane mosaic virus (SCMV) or maize dwarf mosaic virus (MDMV). Frequent tillering and disorders of male and female flowering may also inhibit their use. However, the extreme level of uniformity introduced by artificial selection renders modern elite materials vulnerable to adverse environmental factors and new and emerging pest and diseases [8].

Biotic stress is a major production constraint for maize. Over 38 pests and pathogens, of which Fusarium and Gibberella ear and stalk rots and northern corn leaf blight are the major diseases reported in NW Europe [9]. These diseases gained more importance in NW Europe in the last decade, a development that will continue with increasing temperatures due to climate change [10, 11]. All pathogens and pests together reduce at present grain yield by 22.5% on a global scale in maize [9]. From this, ear and stalk rots together contribute at least 7% to yield gaps. New resistance sources are therefore an important factor to reduce yield gap in hybrid maize production.

Ear rot diseases significantly affect grain production and contaminate grains with mycotoxins [12–14]. In cooler regions such as Europe, northern United States, Canada and some higher altitudes in Africa, Gibberella ear rot (GER) is one of the major types that infect greater proportions of maize [15–20]. GER is caused by the *Fusarium graminearum* species complex, with *F*. *graminearum sensu strictu* Schwabe (teleomorph *Gibberella zeae*) as the most dominant causal agent. This fungus reduces grain weight per ear, hence the yield [12], and contaminates kernels with several mycotoxins of which deoxynivalenol (DON) and zearalenone (ZON) are the most abundant [21]. Up to 48% of yield reduction was reported by Vigier et al. [13] after GER infection in susceptible inbred lines in Canada. Existing management options are mainly related to best agronomic practices, the adoption of mycotoxin reduction technologies, the utilization of fungicides to control pathogens infections [22–24] and resistance breeding [25, 26].

Previous pre-breeding studies revealed a high genetic diversity and the existence of new sources of resistance against GER in European maize landraces [27, 28]. Brauner et al. [29] investigated the performance of testcrosses of DH lines from European flint maize landraces and demonstrated their high potential for the improvement of elite germplasm. Gaikpa et al. [27] reported eight QTL significantly associated with GER resistance in “Kemater Landmais Gelb” (KE, from Austria) landrace. Recently, Akohoue and Miedaner [30] conducted a quantitative trait loci (QTL) meta-analysis which revealed that diverse sources of resistance, including the KE landrace, contributed to the identification of 40 meta-QTL harbouring several putative resistance genes. This demonstrates that the European flint landraces represent important sources of resistance which could be explored for their introgression into elite cultivars. Here we want to test the possibility for using selected lines from landraces for improvement of elite maize for Gibberella ear rot (GER) resistance.

This study aims to (i) select GER-resistant lines from KE and PE landraces to be used as donor parents in crosses with two adapted and highly susceptible flint lines to generate six DH populations, (ii) evaluate the effectiveness of the introgression of GER resistance genes into the adapted lines using donor lines from KE and PE landraces, and (iii) select most GER-resistant lines from each DH population and compare their performance with the two susceptible flint lines. GER severity was our target trait evaluated in the study. Plant height, days to silking, seed-set and plant vigor were recorded only as adaptation traits to evaluate their interaction with GER severity and describe the selected GER-resistant lines from each DH population in comparison with the two adapted susceptible flint lines.

## Materials and methods

### Plant materials and field experiments

Two experiments were followed in this study: (1) Testing of 15 landrace DH lines for their GER resistance in 2021 and 2022, and (2) testing of DH populations derived from crosses of two adapted susceptible flint lines with four donor landrace DH lines.

### Testing of 15 DH lines in 2021 and 2022 (experiment 1)

Eight DH lines from “*Kemater Landmais Gelb*” (KE, from Austria) landrace and seven DH lines from “*Petkuser Ferdinand Rot*” (PE, from Germany) landrace were selected from a previous study by Gaikpa et al. [27] out of 250 lines per landrace. These 15 DH lines were evaluated in Hohenheim (HOH, near Stuttgart) and Gondelsheim (GON, near Karlsruhe) in Germany in 2021 and 2022. Two adapted GER-susceptible flint lines (Flint1 and Flint2) and seven standard checks (Table 1) were also included, making a total of 24 lines which were evaluated at each location using a randomized complete block design (RCBD) with three replicates in 2021 and an alpha lattice design with two replicates in 2022 (see below). Parental and standard lines were therefore included in both experiments, so we had four environments for these materials (2 locations, 2 years). Standard checks CO441 and CO354 were provided by Prof. Lana M. Reid from Eastern Cereal and Oilseed Research Centre, Central Experimental Farm, Agriculture and Agri-Food Canada, Ottawa, Canada [31], while the French dent line F353 was provided by INRAE, Paris, France. Other standard checks such as Check-Dent-res, Check-Dent-sus, Check-Flint-res and Check-Flint-sus, as well as the adapted flint lines (Flint1 and Flint2) were provided by KWS SAAT SE & Co. KGaA. In each experiment, each plot consisted of 20 plants in a single row of 3 m length with an inter-row spacing of 0.75 m and within-row spacing of 0.15 m. From this experiment 1 in 2021, KE3, KE7 and KE8 were selected as GER-resistant DH lines, and PE2 as a moderately GER-resistant DH line by artificial infection.

**Table 1:** Recipient and standard lines included in experiments 1 and 2 and their corresponding Gibberella ear rot (GER) status.

Line	Code	GER status
**Recipient parents:**		
Flint1	Flint1	susceptible
Flint2	Flint2	susceptible
**Standard checks:**		
Check_Dent_res	Dent_res	resistant
Check_Dent_sus	Dent_sus	susceptible
Check_Flint_res	Flint_res	resistant
Check_Flint_sus	Flint_sus	susceptible
CO354	CO354	susceptible
F353	F353	resistant
CO441	CO441	resistant

GER severity was the percentage of a maize ear visually affected by mycelium. All lines were evaluated for GER severity at two locations in 2021 and 2022

### Testing of landrace-derived DH populations in 2022 (Experiment 2)

Six doubled haploid (DH) populations were generated from the following F1 crosses: Flint1×KE3, Flint2×KE3, Flint1×KE7, Flint2×KE7, Flint2×KE8 and Flint1×PE2 with 81, 82, 34, 65, 127 and 145 DH lines, respectively, as outcome of the established method for maize DH production by vivo induction of maternal haploids by a male haploid inducer genotype. These landrace-derived DH populations can also be considered as the first generation of GER resistance introgression into elite lines. KE3, KE7, KE8 and PE2 selected from experiment 1 (2021) were used as GER resistance donor parents, while Flint1 and Flint2 were included as susceptible recipient parents. All materials were multiplied in an off-season program in Chile by KWS SAAT SE & Co. KGaA, Germany.

Afterwards, all the 534 DH lines developed from the six DH populations were evaluated in 2022, together with the 24 parental lines and standard checks from experiment 1 at the same two locations, using an alpha lattice design with two replicates. Plot size was as explained above for experiment 1.

### Artificial inoculations and data collection

Inoculation and data collection were performed similarly for both experiments. The highly aggressive *F*. *graminearum* isolate IFA66 kindly provided by Prof. Dr. Marc Lemmens (University of Natural Resources and Life Sciences, Vienna, Austria) was used to prepare our inoculum suspension following the protocol of Reid et al. [32]. IFA66 was originally isolated by the Department für Agrarbiotechnologie, IFA-Tulln (Tulln an der Donau, Austria) from maize [33]. An aggressiveness test of this isolate (coded as Fg1) was done by Miedaner et al. [34] who revealed that IFA66 was not significantly different from the most aggressive isolates. Exactly 2 mL of the inoculum suspension containing 1.5 x 10^4^ spores.mL^-1^ [27, 35, 36] were applied with a one-needle vaccinator with automatic refill on the silk channel of each cob at 10 plants per row. Artificial inoculations were done five to six days after silk emergence.

All phenotypic traits were recorded for ten maize plants per plot individually. GER severity, which was our target trait, was rated as the percentage of a maize ear visually affected by mycelium, with a score of 0% representing no visible infection and 100% means that all kernels per cob were infected. Additionally, important adaptation traits such as days to silking (DS), plant height (PH, cm), seed-set (SS, %) and plant vigor (PV) were also recorded. DS was recorded when at least 50% of plants per row showed female flowers. SS was collected during disease rating as the percentage of a maize ear covered by kernels. PV was rated 30 to 35 days after sowing at each location on a scale of 1−9 using the height of the plants and color and size of the leaves as criteria, where 1 = very short plants, very small yellowish leaf blades, very poor vigor, and 9 = very tall plants, very large and green leaf blades, excellent vigor [37].

### Data analysis

Basically, we used plot data for each trait. The raw data of the ten plants per plot was first explored to remove outliers per row using the Bonferroni-Holm approach based on re-scaled median absolute deviation (MAD) for standardizing residuals (BH-MADR) described by Bernal-Vasquez et al. [38]. Afterwards, plants with seed-set of 0% were also removed. This resulted in the removal of about 10% of the entire data set. To reduce heterogeneity and ensure the normality of the data, GER severity was transformed using the arcsine square root method [39]. Pearson correlation analysis was conducted between the original GER severity and the transformed values to assess the validity of the transformation.

Furthermore, a two-stage analysis was performed for each trait, following the fully efficient procedure described in detail by Piepho et al. [40] and Buntaran et al. [41]. The procedure is said “fully efficient” because the full variance-covariance matrix of adjusted means in the first stage is forwarded to the second stage of the analysis. According to Buntaran et al. [41], estimates from the fully efficient two-stage analysis are highly correlated (0.97) with the standard single-stage analysis, showing the similar performance of the two types of analysis. Here, the application of the two-stage analysis was relevant for estimating adjusted means of parental and standard lines which were included in the two experimental designs (RCBD and alpha lattice) applied in the study. The data was hierarchized by trial within environment and block.

In the first stage, the data was aggregated at trial level and best linear unbiased estimators (BLUEs) were calculated for the lines following the mixed linear model:

$$\mathrm{RCBD}:\;Y_{ik}=\mu +G_{i}+B_{k}+\varepsilon_{ik}$$ (1)

$$Alpha\;lattice\;design:\;Y{’}_{ikp}=\mu +G_{i}+Rp+B_{pk}+\varepsilon_{ikp}$$ (2)

where *Y*_*ik*_ = response of genotype *i* in block *k; Y’*_*ikp*_ = response of genotype *i* in replicate p and block *k*; *μ* = general mean effect, *G*_*i*_ = genotype, *R*_*p*_ = replicate, *B*_*pk*_ = block nested within replicate, and *ε*_*ik*_ and *ε*_*ikp*_ = residual error. In these models, genotype was fixed while replicate and block were used as random effects. The RCBD equation applied to the experiment evaluating the parents and standard lines in 2021 (experiment 1), while the alpha lattice equation analyzed the experiment evaluating the parents, standard lines in 2022 (experiment 1) and the six DH populations in 2022 (experiment 2).

In the second stage, a mixed linear model was fitted across environments using the BLUEs and the full variance-covariance matrix from the first stage to estimate variance components and broad sense heritability for each trait as follows:

$$Y_{i}{}_{l}=\mu +G_{i}+E_{l}+GE_{i}{}_{l}+\varepsilon_{il}$$ (3)

where *Y*_*il*_ = BLUE of genotype *i* within environment *l*; *μ* = general mean effect, *G*_*i*_ = genotype *i* within environment *l*, *E*_*l*_ = environment, *GE*_*il*_ = genotype by environment interaction and *ε*_*il*_ = residual error associated with BLUE of genotype *i* within environment *l*. Variance-covariance matrix was used to separate genotype-environment interaction and the residual variances.

Dummy variables (0, 1) were used to separate populations, donor and recipient parents [42]. For each population, in defining the dummy variable, 1 was applied for all lines belonging to the population and 0 for others lines. The interaction between genotype and each dummy variable (*Dummy*:*genotype*) generated estimates for all lines coded as 1. Therefore, this interaction term was applied to subset the data and estimate variance components for each population separately. Genotype, environment and genotype-environment effects were used as random in the second stage model to estimate variance components. The likelihood ratio test (LRT) was implemented to analyze significance of variance components [43].

Broad sense heritability (H^2^) was estimated as follows [44, 45]:

$$H^{2}=\left(1‐\frac{{\bar{v}}_{\Delta \ldots }^{\mathrm{BLUP}}}{{2\sigma }_{g}^{2}}\right)$$ (4)

where $\sigma_{g}^{2}$ is the genotypic variance, ${\bar{v}}_{\Delta }^{\mathrm{BLUP}}$ is the average standard error of difference of two genotypic best linear unbiased predictions (BLUP). Outlier removal, the two-stage analysis and heritability estimates were performed using ASReml v.4.1 [46].

The genotypic coefficient of variation (CV_G_, %) was calculated for each trait as:

$${\mathrm{CV}}_{G}=\left(\frac{\sqrt{\sigma_{g}^{2}}}{\mathrm{Mean}}\right)\;x\;100$$ (5)

where $\sigma_{g}^{2}$ is the genotypic variance. In addition, coefficient of variation due to error (CV_ε,_ %) was estimated by replacing the genotypic variance ($\sigma_{g}^{2}$) by the residual variance ($\sigma_{\varepsilon }^{2}$) in Eq 5.

Based on the BLUEs for GER severity (S1 Table), five best DH lines were selected within each DH population, and their GER severity and BLUEs for adaptation traits were compared to those of the recipient parents and the standard checks. All analyses were conducted in the R software v4.1.0 [47].

## Results

### Frequency distributions and correlations among GER severity and adaptation traits (experiment 1 + 2)

**Fig 1:**
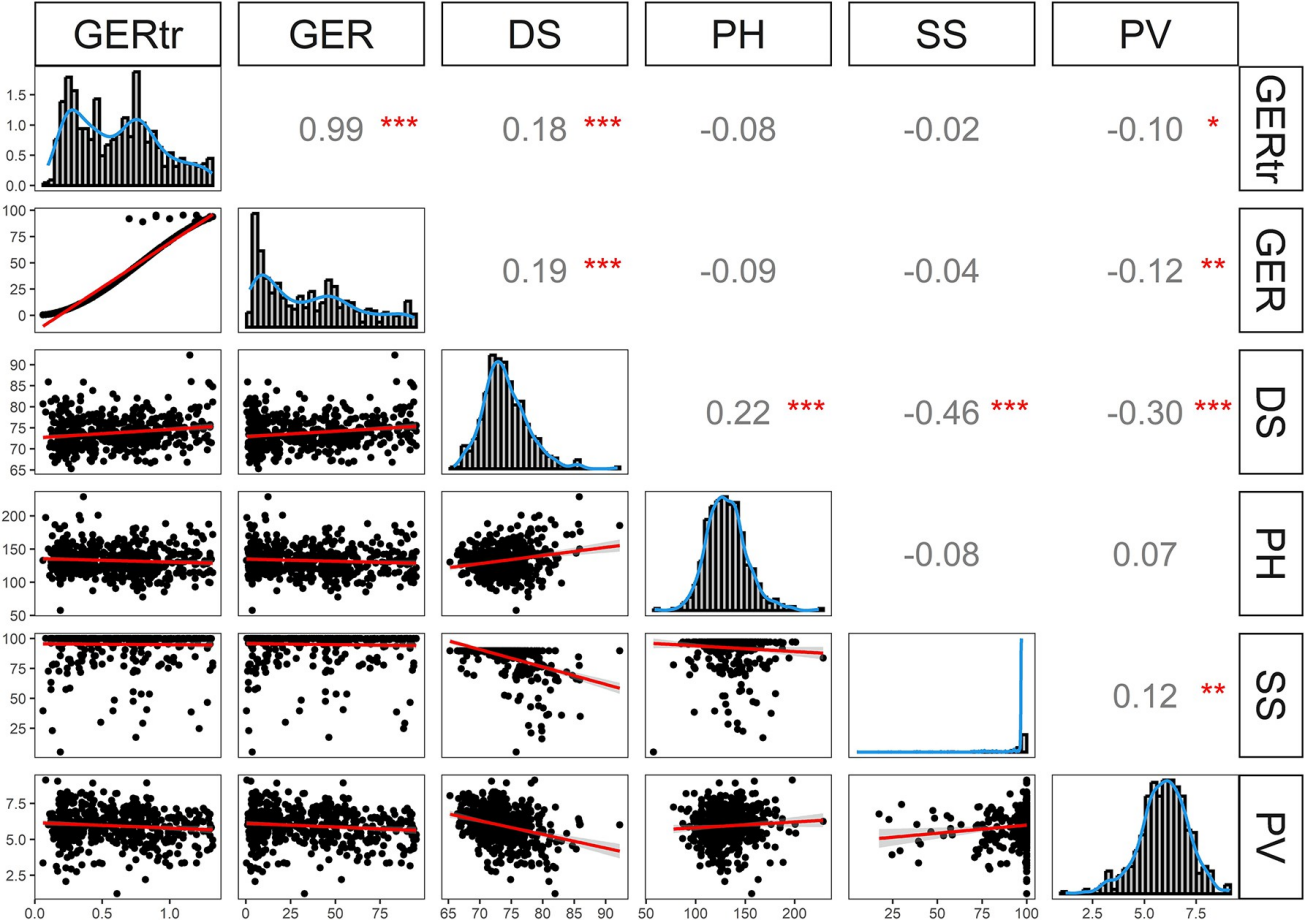
Frequency distributions and correlations among Gibberella ear rot (GER) severity (original values, %), transformed GER severity (GER_tr_), plant height (PH, cm), days to silking (DS), seed-set (SS, %) and plant vigor (PV) based on best linear unbiased estimations of the 15 landraces lines, two recipient parents, nine standard checks and 534 doubled haploid lines from the six populations. *, ** significant at p<0.05, 0.01, respectively.

For illustrating the genotypic variation across both experiments, we firstly present the analysis of the histograms based on best linear unbiased estimations across environments (Fig 1). GER severity and the adaptation traits were quantitatively distributed; with the exception of SS (Fig 1). Most lines exhibited relatively high seed-set across landraces and DH populations. Lines from both landraces have been previously selected for seed-set by Gaikpa et al. [27], and the adapted flint lines had high seed-set (>95%).

Low phenotypic correlations (−0.04 to 0.19) were observed between GER severity and the adaptation traits (Fig 1). Significant negative correlation (−0.46) was detected between DS and SS, which might be due to the increased average temperature recorded in 2022 in all environments; that might lead to seed abortion in late lines. A highly significant correlation was found between original and transformed GER severity, confirming the reliability of the inferences based on the arcsine square root transformation applied in our study. It is of particular interest that the correlation between GER and PV is of low importance, an indication that the low plant vigour of part of the lines does not have a strong effect on *F*. *graminearum* infection.

### Genetic variation of GER severity and adaptation traits within 15 DH lines from landraces (experiment 1)

Within lines drawn from both KE and PE landraces, considerable genetic variation was observed for all traits (Table 2). Genotype by environment interaction variances for GER severity were significant and represented about 1% and 54% of the genotypic variance for KE and PE lines, respectively. Broad sense heritability ranged from 0.62 to 0.98, with the lowest value observed for seed-set (SS) (Table 2). Heritability estimate and genetic coefficient of variation (CV_G_) of GER severity were considerably higher in lines from KE landrace than that of PE landrace lines. The coefficient of variation due to error (CV_ε_) was low (0.78−24.10) for all traits and lower than the CV_G_, except for GER severity of PE landrace (Table 2).

**Table 2:** Descriptive statistics, variance components and heritability estimates of adaptation traits and Gibberella ear rot (GER) severity of 15 lines drawn from Kemater and Petkuser landraces.

Trait	Unit	CV_G_	CV_ε_	$\sigma_{G}^{2}$	$\sigma_{\mathrm{GE}}^{2}$	$\sigma_{\varepsilon }^{2}$	H^2^
**Kemater landrace:**							
GER	%	65.37	24.10	20.60	0.20	2.80	0.98
PH	cm	13.38	2.76	490.90	164.30	20.86	0.93
DS	days	3.09	0.78	6.23	3.92	0.40	0.89
SS	%	6.27	4.42	27.37	8.13	20.41	0.62
PV	-	11.26	6.14	0.47	0.33	0.14	0.63
**Petkuser landrace:**							
GER	%	8.88	17.42	1.10	0.60	4.30	0.76
PH	cm	10.41	3.44	234.90	192.50	25.67	0.86
DS	days	4.15	0.84	11.57	1.97	0.48	0.95
SS	%	11.12	7.51	67.49	15.22	30.78	0.67
PV	-	15.37	7.79	0.78	0.31	0.20	0.67

PH = plant height, DS = days to silking, SS = seed-set, PV = plant vigor, CV_G_ = genotypic coefficient of variation (%), CV_**ε**_ = coefficient of variation of error (%), $\sigma_{G}^{2}$ = genotypic variance, $\sigma_{\mathrm{GE}}^{2}$ = genotype x environment interaction, $\sigma_{\varepsilon }^{2}$ = residual variance, H^2^ = broad sense heritability. The lines were evaluated at two locations in 2021 and 2022. All CV, variance, and heritability estimates were computed with arcsine transformed GER values, variance components of GER severity were multiplied by 100 and minimum, maximum and mean were estimated from back-transformed best linear unbiased estimations (BLUEs) of GER

**Fig 2:**
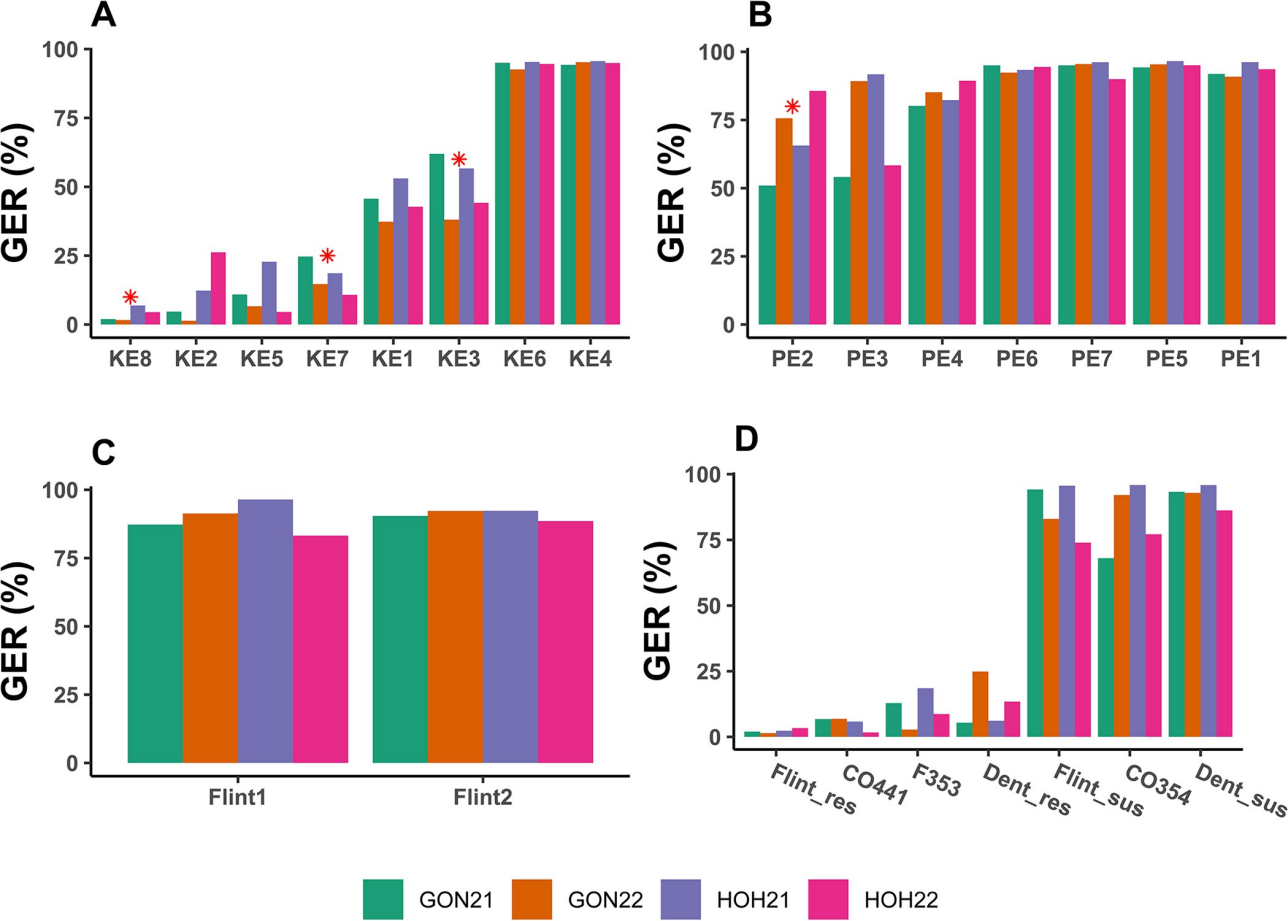
Barplots showing Gibberella ear rot (GER) severity (back-transformed values, %) of parental and standard lines evaluated at each of two locations (GON and HOH) in 2021 and 2022. GON21, GON22, HOH21, HOH22 are the four environments based on location × year combinations. (**A**) doubled haploid lines of Kemater (KE) landrace used as donor parents, (**B**) doubled haploid lines of Petkuser (PE) landrace used as donor parents, (**C**) recipient parents and (**D**) standard lines (res = resistant, sus = susceptible). The asterisk (*) indicates lines selected as donor parents for development of doubled haploid populations.

In 2021, GER severity was low to moderate (2.0−62.0%) at both locations in six KE DH lines such as KE8, KE2, KE5, KE7, KE1 and KE3 (Fig 2A, S2 Table). In contrary to lines from KE landrace, GER severity was higher (>50%) in all lines drawn from PE landrace (Fig 2B, S2 Table). The lowest GER severity was observed with line PE2 within this landrace. GER severity was high (87.3−96.5%) for the adapted parents Flint1 and Flint2 (Fig 2C, S2 Table). Resistant standard lines included in the experiments showed very low to low GER severity (1.4−18.5%), while susceptible standards were highly infected (68.0−95.9%) (Fig 2D, S2 Table).

In 2022, similar ranges were observed for GER severity in both KE and PE DH lines, the adapted parents Flint1 and Flint2, and standard checks. Based on this result, KE8, KE7, KE3 and PE2 which were highly to moderately resistant to GER disease across locations and years, and were selected and crossed with the susceptible adapted parents Flint1 and Flint2 to generate the six DH populations.

### Genetic variation of GER severity within six doubled haploid populations from crosses with elite maize (experiment 2)

Based on the transformed GER severity, significant genotypic and genotype by environment interaction variances were observed within the six DH populations (Table 3). The genotypic variance was 1.4−1.8-fold higher than the genotype by environment interaction in Flint2×PE2, Flint2×KE8 and Flint2×KE7. Depending on the population, the genotypic coefficient of variation was relatively high in all populations, with the exception of Flint1×KE7. Coefficient of variation due to error was low (<20%) for all populations and lower than CV_G_, except for Flint1×KE7 (Table 3). The broad sense heritability estimates were moderate (0.55) to high (0.83), with the lowest value observed in Flint1×KE7 and the highest in Flint2×KE8 (Table 3).

**Table 3:** Variance components, heritability estimates of arcsine transformed Gibberella ear rot (GER) severity of doubled haploid lines within six landrace-derived populations evaluated at two locations in 2022.

Population	Descriptive statistics					Variances and heritability			
	Min	Mean	Max	CV_G_	CV_ε_	$\sigma_{G}^{2}$	$\sigma_{\mathrm{GE}}^{2}$	$\sigma_{\varepsilon }^{2}$	H^2^
DH(Flint1×KE3)	1.55	36.71	96.60	34.35	6.87	5.00	5.30	0.20	0.64
DH(Flint1×KE7)	1.56	26.97	68.04	8.19	14.19	0.20	8.40	0.60	0.55
DH(Flint2×KE3)	1.61	36.58	98.99	30.72	6.85	4.00	5.50	0.20	0.62
DH(Flint2×KE7)	1.50	29.42	96.77	43.78	9.55	6.30	3.60	0.30	0.68
DH(Flint2×KE8)	1.30	33.06	96.02	42.25	5.16	6.70	4.20	0.10	0.83
DH(Flint2×PE2)	1.85	50.95	97.44	35.80	5.63	8.10	5.60	0.20	0.70

Min = minimum, Max = maximum, CV_G_ = genotypic coefficient of variation, CV_**ε**_ = coefficient of variation of error, $\sigma_{G}^{2}$ = genotypic variance, $\sigma_{\mathrm{GE}}^{2}$ = genotype x environment interaction, $\sigma_{\varepsilon }^{2}$ = residual variance, H^2^ = broad sense heritability. All CV, variance, and heritability estimates were computed with arcsine transformed GER values, variance components of GER severity were multiplied by 100 and minimum, maximum and mean were estimated from back-transformed best linear unbiased estimations (BLUEs) of GER

### Comparison of GER severity of landrace-derived DH populations with recipient parents and performance of the five best lines within each population (experiment 2)

**Fig 3:**
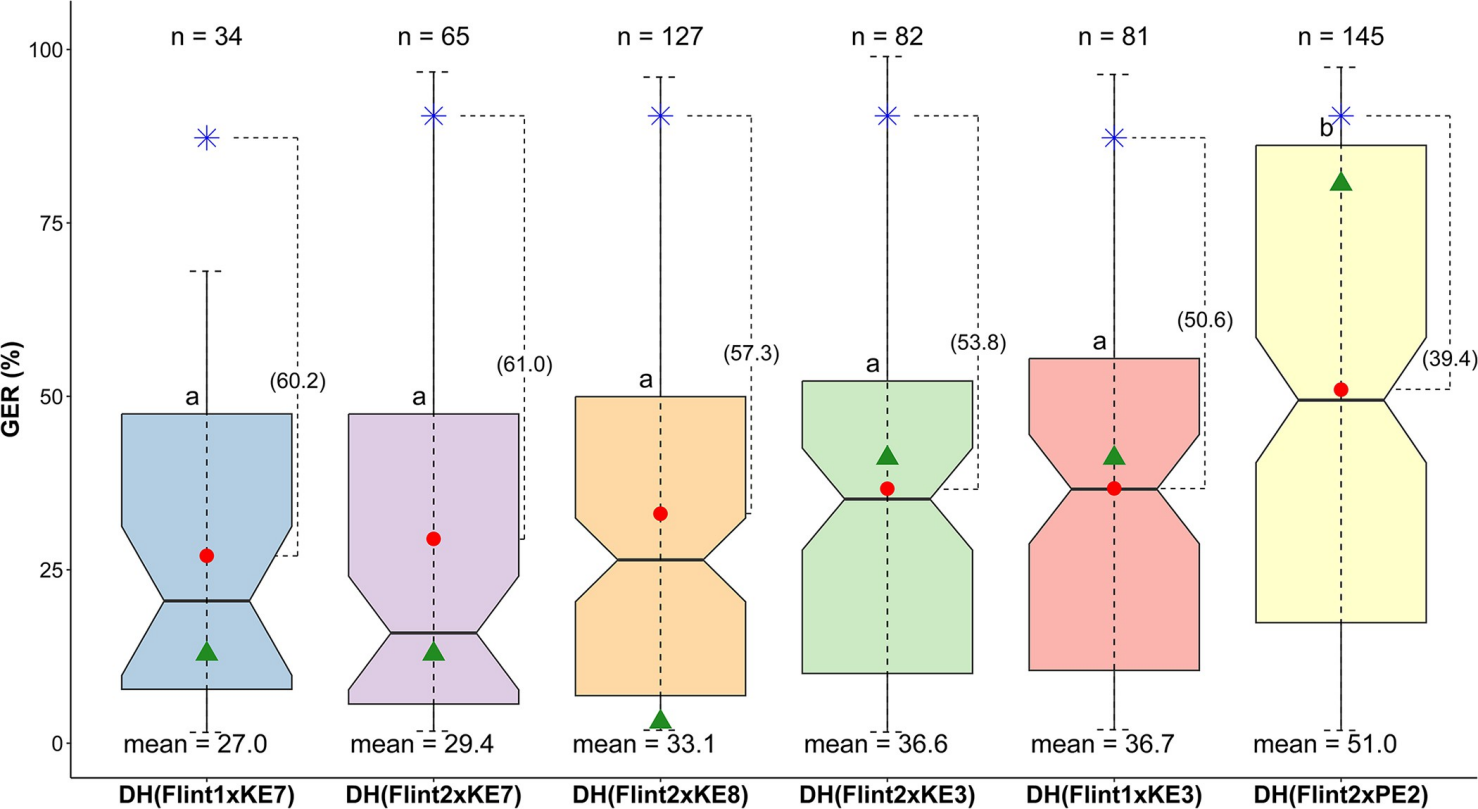
Boxplots showing Gibberella ear rot (GER) severity (back-transformed values, %) of DH populations evaluated at two locations in 2022. n indicates the number of doubled haploid lines analyzed per population. Horizontal lines within boxes indicate the median. For each box, the notch represents 95% confidence interval for the median. Red point, green triangle in each box and blue asterisk (*) indicate mean GER severity of each DH population, donor parent and recipient parent, respectively. Values in parenthesis are the differences between population mean and mean GER severity of the corresponding recipient parent. Boxes with the same letter are statistically identical at p<0.05.

Based on mean GER severity, significant difference was observed between KE-derived populations and PE-derived population (Fig 3). No significant differences were observed among the five KE-derived populations. Flint2×PE2, a PE-derived population exhibited the highest average GER severity (51.0%), while lower GER severity (27.0−36.7%) was observed in KE-derived DH populations, although both recipient flint lines were similarly susceptible (Fig 3). In addition, on average, the six DH populations were more resistant than the recipient parents (Fig 3). In comparison to the mean performance of Flint1 (87.2%) and Flint2 (90.4%) across the two locations in 2022, average GER severity was reduced by 39.4−61.0% in the DH populations. GER severity reduction was higher in all KE-derived populations (53.6−61.0% reduction) than the PE-derived population (39.4% reduction).

From the six DH populations, the five best lines were very resistant, with mean GER severity of 17−168-fold lower than mean GER severity of Flint2 and Flint1 (Table 4). Furthermore, GER severity of selected lines were similar to that of Flint_res which was the most resistant standard check included in the study. Regardless of the populations, the selected lines exhibited similar performances for all adaptation traits, except for average SS which was relatively low for lines 101 (39.5%) and 283 (23.0%) from Flint1×KE7 and Flint2×KE7, respectively (Table 4). Average PV was also relatively low for lines 101 (3.3) and 501 (3.1) from Flint1×KE7 and Flint2×PE2. In comparison with the recipient parents, average DS and PV of selected lines were similar in the six populations. However, most selected lines were relatively shorter in all populations than the recipient parents.

**Table 4:** Performances of the five best doubled haploid (DH) lines within each population in comparison with recipient parents and standard checks evaluated at two locations in 2022.

Population	Line	GER (%)	DS (days)	PH (cm)	SS (%)	PV (1–9)
DH(Flint1×KE3)	70	1.55	70.29	133.80	94.96	6.54
DH(Flint1×KE3)	45	1.60	76.27	139.95	93.74	5.31
DH(Flint1×KE3)	87	1.65	71.20	131.33	97.74	6.77
DH(Flint1×KE3)	67	1.95	70.65	122.55	96.99	6.28
DH(Flint1×KE3)	19	2.86	71.88	159.27	90.99	9.04
DH(Flint1×KE7)	101	1.56	80.64	132.93	39.54	3.34
DH(Flint1×KE7)	107	2.40	75.79	155.80	57.43	5.05
DH(Flint1×KE7)	110	3.57	69.32	152.80	92.96	6.48
DH(Flint1×KE7)	114	4.35	70.50	156.13	90.91	6.32
DH(Flint1×KE7)	93	5.20	67.62	145.86	89.99	7.85
DH(Flint2×KE3)	145	1.61	70.44	110.00	96.83	6.76
DH(Flint2×KE3)	142	2.23	66.69	147.76	97.74	5.91
DH(Flint2×KE3)	150	2.49	74.25	123.46	99.50	6.37
DH(Flint2×KE3)	175	2.86	67.35	114.08	93.91	5.44
DH(Flint2×KE3)	132	3.21	70.23	128.97	98.00	6.93
DH(Flint2×KE7)	250	1.50	74.55	144.39	57.43	6.47
DH(Flint2×KE7)	219	1.70	78.72	197.65	99.00	9.12
DH(Flint2×KE7)	283	1.88	78.16	147.75	22.98	6.38
DH(Flint2×KE7)	284	1.88	73.37	118.95	97.00	6.15
DH(Flint2×KE7)	254	1.95	76.22	145.20	78.61	5.02
DH(Flint2×KE8)	363	1.30	71.39	145.60	94.99	5.73
DH(Flint2×KE8)	364	1.68	71.50	125.08	96.02	6.23
DH(Flint2×KE8)	315	1.88	68.22	131.33	95.74	6.27
DH(Flint2×KE8)	317	1.88	70.67	105.61	96.99	5.54
DH(Flint2×KE8)	327	1.88	77.62	169.92	77.78	6.79
DH(Flint2×PE2)	566	1.85	85.88	150.00	72.61	6.00
DH(Flint2×PE2)	460	2.21	72.05	105.95	94.83	7.77
DH(Flint2×PE2)	561	2.21	73.40	113.68	96.99	6.26
DH(Flint2×PE2)	501	2.54	81.67	125.55	78.61	3.11
DH(Flint2×PE2)	568	3.21	71.34	129.58	95.01	7.46
Recipient	Flint1	87.26	74.64	165.40	95.33	7.16
Recipient	Flint2	90.42	76.50	169.90	96.99	6.26
Standard checks	Flint_res	2.39	186.90	81.97	97.93	6.38
Standard checks	Dent_sus	89.55	187.50	85.71	97.83	6.43
-	Gross mean	9.95	80.10	135.70	87.47	6.32
-	LSD 5%	7.30	5.57	14.08	22.15	5.01

GER = Gibberella ear rot severity (back-transformed values), PH = plant height, DS = days to silking, SS = seed-set, PV = plant vigor (1 = no vigour, 9 = highest vigour), LSD 5% = Fisher’s least significant difference at 5% significance level

## Discussion

The exploitation of European landrace germplasm could be of paramount importance for developing elite materials with improved Gibberella ear rot resistance. We selected three DH lines from Kemater landrace and one DH line from Petkuser landrace, which had varying degrees of GER resistance and used them as donors to develop six DH populations to improve GER resistance of two adapted flint lines (Flint1 and Flint2) included as recipient parents. The six DH populations with a total of 534 DH lines were on average 39.4−61.0% less infected than the mean performance of the adapted flint lines. Within each DH population, the performance of five most resistant DH lines from each population was discussed for consideration in elite maize breeding programs. We will also include results from Gaikpa et al. [27] to discuss the relative stability of GER resistance of the 15 KE and PE lines evaluated in this study.

### Genetic variation and heritability estimates of GER severity within lines from landraces and landrace-derived DH populations

Genotypic variances ($\sigma_{G}^{2}$) within DH lines from KE landrace were generally much higher than within DH lines drawn from PE landrace and were further reduced in derived DH populations. Similarly, heritability was higher in KE landrace lines (0.98) than PE landrace lines (0.76), and in both KE- and PE-derived DH populations (0.55−0.83). The remarkably high entry-mean heritability (0.98) detected in the study indicates that GER is a highly heritable trait, but also highlights an appropriate field phenotyping as indicated by Piepho and Möhring [45]. Gaikpa et al. [27] who evaluated 500 DH lines from both KE and PE landraces reported similar high heritability estimates (0.80 for KE and 0.77 for PE) for GER severity. Furthermore, heritability observed within the six DH populations included in our study confirms the results of Galiano-Carneiro et al. [35] who reported similar values (0.24−0.72) when evaluating six Brazilian donor-derived DH populations across environments in Brazil and Europe. The existence of significant genetic variation observed in our study indicates that the DH populations can be used in QTL mapping studies to identify and validate resistance loci for accelerating breeding for GER resistance in elite maize materials.

The genotype by environment interaction ($\sigma_{\mathrm{GE}}^{2}$) was also significant within lines from both KE and PE landraces and derived DH populations, even though it was lower than the genotypic variance. Significant genotype by environment interactions in this pathosystem were also reported by Akohoue et al. [36], Gaikpa et al. [27] and Bolduan et al. [48]. The relative magnitude of $\sigma_{G}^{2}$ compared to $\sigma_{\mathrm{GE}}^{2}$ was higher within landrace lines than the derived DH populations, revealing that the contribution of genotype by environment interaction to GER resistance was more important in the DH populations.

### Differential GER resistance of European flint landraces

Our study demonstrated that mean GER severity of the seven PE DH lines was on average about 2.1-fold higher than GER severity of the eight KE DH lines across the four environments. This demonstrates a clear superiority of lines from “*Kemater Landmais Gelb*” landrace over “*Petkuser Ferdinand Rot*” landrace lines with regards to resistance to Gibberella ear rot in maize. Gaikpa et al. [27] evaluated 250 DH lines from each landrace and found that average GER severity within PE landrace was 1.3-fold higher than that within KE landrace. Similarly, Akohoue et al. [36] reported that GER severity of PE DH lines evaluated in four environments in Germany was 1.3-fold higher that KE DH lines. Furthermore, based on genome-wide association study (GWAS) of 250 KE DH lines, Gaikpa et al. [27] reported eight GER resistance loci with individual additive effect size of −3.27 to −5% and a total of 33.69% of genotypic variance explained. In contrary to the KE landrace, no significant marker-trait associations were identified for GER severity within PE landrace using the GWAS approach. This firstly demonstrates that GER resistance is quantitatively inherited with both additive and dominance effects as reported by Butrón et al. [49], Martin et al. [50] and Mesterházy et al. [51]. Although additive gene action is predominant, Martin et al. [50] also found significant dominance effects in a cross (D152×UH007) for GER resistance and in four crosses for DON contamination (D152×UH006, D152×UH007, UH007×UH006, UH009×UH006). Secondly, the absence of significant marker-trait associations reported within the PE landrace confirms that QTL effects of GER resistance within this landrace might be too low to be exploited through marker-assisted selection.

**Fig 4:**
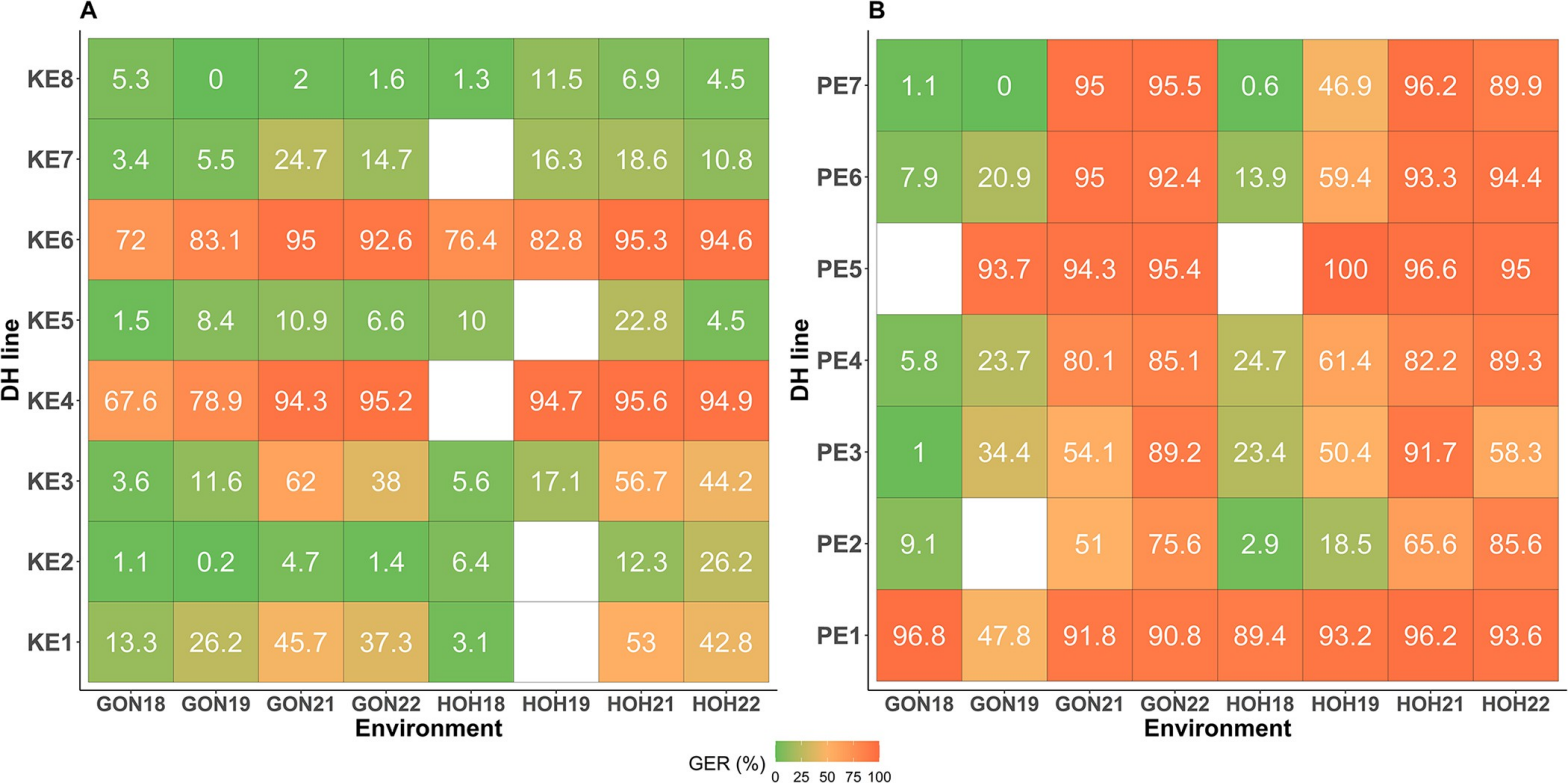
Heatmaps showing relative stability of mean Gibberella ear rot (GER) severity (back-transformed values, %) of lines drawn from landraces evaluated in eight environments (= two locations × four years). (**A**) GER severity of eight doubled haploid (DH) lines from Kemater landrace and (**B**) GER severity of seven DH lines from Petkuser landrace. Color gradient shows the relative severity and stability of GER infections in each environment. Mean GER severity in 2018 and 2019 were reported by Gaikpa et al. [27].

Moreover, the average GER severity of KE DH lines (40.94%) observed in the present study was similar to GER severity of 44.12% and 42.13% reported for DH lines from the same landrace population by Gaikpa et al. [27] and Akohoue et al. [36], respectively. Lines KE1, KE2, KE3, KE5, KE7 and KE8 which were moderately to highly resistant in this study were also identified by Gaikpa et al. [27] as resistant genotypes across four different environments (Fig 4). A compilation of our results with those of Gaikpa et al. [27] for the same landrace lines revealed that resistant lines KE2, KE5, KE7 and KE8 maintained a relatively low mean GER severity (<30%) across all environments, while KE1 and KE3 were more unstable with GER severity of 3.6−62% (Fig 4A). This demonstrates a relative stability of GER resistant KE landrace lines across environments. In contrary to KE landrace, mean GER severity of PE landrace lines in our study (86.19%) was 1.5−1.6-fold higher than that reported by Gaikpa et al. [27] (58.57%) and Akohoue et al. [36] (55.04%). In addition, mean GER severity of PE lines were highly variable (0–85.6%) across environments (Fig 4B). This reveals that the reaction of the PE landrace lines to GER infection across environments was less stable than that of the KE landrace lines. In contrary to moderately resistant lines, susceptible lines (KE4, KE6, PE1 and PE5) were stable with very high GER severity in all environments. Stability of GER resistance was also investigated by Dalla Lana et al. [52] who evaluated 15 maize hybrids to Gibberella ear rot across 30 environments (= 3 years × 10 locations) in the USA, and found also differences in the stability according to the resistance level. Butrón et al. [49] also reported a low stability of GER resistance when evaluating two maize crosses (CO359 × CO441 and EP42 × EP77) across four environments in Canada and Spain. This emphasizes the need for evaluating landrace lines across multiple environments in order to select most resistant and stable lines which could be used as donor parents in breeding programs. Environment-specific GER resistance breeding targets should also be defined for a better and more efficient exploitation of the large existing genetic diversity within landraces. In addition, with recent advances and availability of molecular genomic information on the trait [27, 30, 53, 54], genomics-assisted selection could be applied for accelerated evaluation for GER resistance in maize.

### Effectiveness of GER resistance introgression into elite inbreds

On average, GER severity of the DH populations was 39−61% lower than that of the two adapted elite lines (Fig 3). This is an indication that the selected donor landrace lines were effective in improving GER resistance of the adapted elite inbreds. In addition, the finding confirms the usefulness of selected donor landrace lines for improving the resistance of European adapted flint lines as demonstrated by Galiano-Carneiro et al. [35] using Brazilian tropical DH lines as donor parents.

Moreover, the PE-derived DH population was significantly more susceptible than the KE-derived populations. This, once again confirms the superiority of KE landrace donors for transferring GER resistance into elite materials.

The five best DH lines selected within each populations were 17−168-fold more resistant than the elite lines. The lines exhibited similar average performances with the elite lines for days to flowering, seed-set and plant vigor, with the exception of lines 101 and 283 from Flint1×KE7 and Flint2×KE7, respectively, which had a lower seed-set (Table 4). This shows that they are already adapted according to DS, PH, SS, and PV; however, this should be validated through further evaluations especially for grain and stover yield. This is not astonishing because the landraces were innately adapted to the eco-climatic conditions of NW Europe. In addition, the selected lines showed similar GER resistance compared to the most resistant standard check included in the study. This may be explained by the high genetic diversity observed within the DH populations where the best lines exhibit very low GER severity (<10%), while the most susceptible lines show very high GER severity across environments. This exhibits the high potential of the six DH populations evaluated in this study of which the best lines could be integrated into elite breeding programs for improving GER resistance. However, best lines from these populations should be further evaluated through multi-location and multi-year trials to select the most stable lines to be recommended for improved and sustainable hybrid maize production. In addition, it is also important to evaluate the combining ability of the selected lines for grain yield and GER resistance, their resistance to other ear rot pathogens (i.e. *F*. *verticillioides*) as well as their response to mycotoxin contaminations.

## Conclusions

Understanding the effectiveness of introgression of GER resistance from landraces into elite materials is required to better exploit the potential of European flint landraces. The use of KE landrace as donor lines was more effective for reducing GER severity of susceptible elite lines than PE landrace. Our results showed that the derived DH populations are genetically diverse and of high interest for use in maize breeding programs. The introgression of GER resistance genes into elite materials could be accelerated through the application of genomics-assisted selection within populations. Considering the complexity and quantitative nature of GER resistance, backcross selection could be combined with genomic selection and prediction which seem to be the most relevant molecular approaches that could be implemented within each population to rapidly improve GER resistance of elite materials as indicated by Akohoue and Miedaner [30]. Given the existence of significant genotype by environment interaction within the DH populations, the integration of genomics-assisted breeding for increased selection efficiency could be implemented within environment to better exploit QTL by environment interaction and define area-specific breeding targets. The selected lines from each DH population had similar GER resistance than the highly resistant standard line included in the study. These best lines could be incorporated into breeding programs across multiple locations and years to select the most stable lines.

## Supporting information

S1 Table Best linear unbiased estimations (BLUEs) of Gibberella ear rot (GER) severity (back-transformed values, %), days to silking (DS, days), plant height (PH, cm), seed-set (SS, %), and plant vigor (PV) across environments. Doubled haploid (DH) populations were evaluated across two locations, while others lines were evaluated across four environments.
(XLSX)

S2 Table Back-transformed best linear unbiased estimations (BLUEs) of Gibberella ear rot (GER) severity of parental and standard lines evaluated at two locations (GON and HOH) in 2021 and 2022. 4_ENV = mean of GER severity (back-transformed values, %) across the four environments, LSD 5% = Fisher’s least significant difference at 5% significance level. GER severity was the percentage of a maize ear visually affected by mycelium.
(XLSX)
